# Immunology Guides Skeletal Muscle Regeneration

**DOI:** 10.3390/ijms19030835

**Published:** 2018-03-13

**Authors:** F. Andrea Sass, Michael Fuchs, Matthias Pumberger, Sven Geissler, Georg N. Duda, Carsten Perka, Katharina Schmidt-Bleek

**Affiliations:** 1 Julius Wolff Institut, Charité—Universitätsmedizin Berlin, 13353 Berlin, Germany; andrea.sass@charite.de (F.A.S.); michael.fuchs2@charite.de (M.F.); sven.geissler@charite.de (S.G.); georg.duda@charite.de (G.N.D.); katharina.schmidt-bleek@charite.de (K.S.-B.); 2 Berlin-Brandenburg Center for Regenerative Therapies, Charité—Universitätsmedizin Berlin, 13353 Berlin, Germany; carsten.perka@charite.de; 3 Center for Musculoskeletal Surgery, Charité—Universitätsmedizin Berlin, 13353 Berlin, Germany

**Keywords:** healing, inflammation, macrophage, scaffold, angiogenesis, growth factors

## Abstract

Soft tissue trauma of skeletal muscle is one of the most common side effects in surgery. Muscle injuries are not only caused by accident-related injuries but can also be of an iatrogenic nature as they occur during surgical interventions when the anatomical region of interest is exposed. If the extent of trauma surpasses the intrinsic regenerative capacities, signs of fatty degeneration and formation of fibrotic scar tissue can occur, and, consequentially, muscle function deteriorates or is diminished. Despite research efforts to investigate the physiological healing cascade following trauma, our understanding of the early onset of healing and how it potentially determines success or failure is still only fragmentary. This review focuses on the initial physiological pathways following skeletal muscle trauma in comparison to bone and tendon trauma and what conclusions can be drawn from new scientific insights for the development of novel therapeutic strategies. Strategies to support regeneration of muscle tissue after injury are scarce, even though muscle trauma has a high incidence. Based on tissue specific differences, possible clinical treatment options such as local immune-modulatory and cell therapeutic approaches are suggested that aim to support the endogenous regenerative potential of injured muscle tissues.

## 1. Clinical Background

Skeletal muscle belongs to the largest organ system in the human body, accounting for one-third of body weight [1]. Its function is essential for the musculoskeletal system, which is responsible for coordinated movement and postural maintenance, as well as key functions in metabolism and energy homeostasis. The importance of skeletal muscle becomes apparent when its function is impaired due to injury. A major problem is the formation of structurally and biologically altered tissue: fibrotic scar tissue, which develops if a trauma exceeds the intrinsic regenerative capacity of the muscle. In this context, scar tissue formation can be considered as regeneration failure as it never gains full functional integrity.

Iatrogenic skeletal muscle injuries are an inevitable result of almost any surgical approach, particularly in orthopedic surgery. Hundreds of thousands of surgeries are performed for the hip joint or lumbar spine each year, and the rates are predicted to increase [2,3,4]. A lack of muscular regeneration is a common cause for impeded mid- and long-term results after total hip or knee arthroplasty [5,6,7]. For example, the gluteus medius muscle is frequently damaged during total hip arthroplasty, which can lead to joint dislocation as well as pelvitrochanteric insufficiency and thus to poor function and patient-reported long-term outcome scores [8,9]. 

Up to 20% of patients develop an abductor deficiency after total hip arthroplasty [10]. This is especially true for older patients due to a reduced regenerative capacity resulting from higher muscle atrophy [11]. Minimally invasive approaches intend to reduce soft tissue damage. However, it has been shown that the skeletal muscles of the pelvic and spinal column are significantly impaired in its function even when minimally invasive interventions with smaller incisions and thus a limited exposition of the surgical field were performed [12,13]. Despite this ubiquitous problem, there is currently no causative treatment available for either sport-related or iatrogenic skeletal muscle injuries. 

## 2. Muscle Regeneration

The processes underlying muscle regeneration have been investigated for over 150 years [14], and it is well-established that skeletal muscle holds an innate capacity to regenerate. If the injury is minor, the healing process leads to normal, integer, and functional tissue.

However, if the extent of the injury exceeds the regenerative capacity of the tissue, it results in a loss of contractile muscle tissue and the development of fibrosis. Moreover, the injury location needs to be taken into consideration. Hamstring strain muscle injuries occur with high incidence rates and are an example for slow healing and persisting symptoms, with almost one third still showing signs of injury one year after the damage with high re-injury risks [15]. 

### 2.1. Inflammation Onset, Development, and Resolution

For muscle tissue healing, inflammatory processes are required in order to reduce the size of necrotic or damaged areas and to replace them with new tissue. Also, the flow onset, development, and resolution of inflammation plays a critical role on the regulation of satellite cell function and therefore muscle regeneration. 

Tissue injury is initially accompanied by bleeding from damaged vessels. Bleeding triggers coagulation, and a haematoma is formed as a first step in healing [16,17,18]. The clotting reaction is coupled with a pro-inflammatory reaction [19]. Small molecules called anaphylatoxins are formed in the periphery of the injury that activate sentinel cells such as mast cells [20], which further trigger the inflammatory response and stimulate the proliferation of satellite cells [21]. In addition, intracellular proteins and molecules from the extracellular matrix are released from the damaged tissue. They activate resident cells at the site of injury [22], promoting the early inflammatory reaction. Due to the absence of a functional vasculature at this stage, sufficient oxygen supply cannot be maintained, which leads to a hypoxic atmosphere with decreased pH values, as it is known from bone healing [18,23,24,25,26]. Under these conditions, only selected immune cells are capable of switching to an anaerobic metabolism in order to survive and trigger the necessary inflammatory response through cytokine and chemokine secretion. While T-cells can tolerate the low oxygen levels in the early haematoma for a while and macrophages can quickly adapt and switch to anaerobic glycolysis [27], the local anaerobic milieu with low pH values is challenging for the survival of endothelial progenitor cells [23]. However, angiogenic processes and hence revascularization are crucial to initiate a regenerative cascade. To support (endothelial) progenitor cell survival and proliferation, the more robust immune cells must initiate re-vascularization via cytokine signalling in order to further the healing process [23]. 

**Figure 1:**
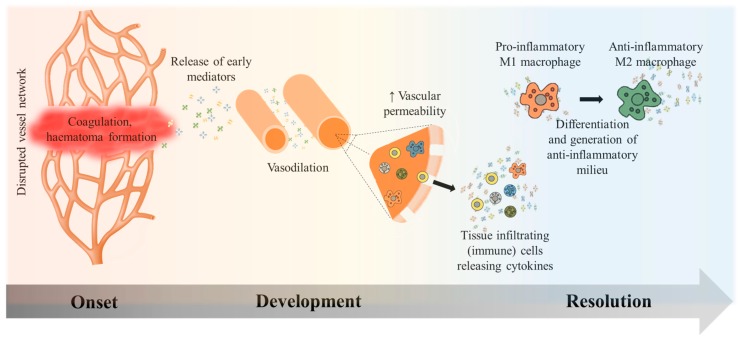
Course of inflammation in skeletal tissue regeneration. Signalling molecules released from the early haematoma result in vascular dilation and increased permeability. Circulating cells from the periphery can now enter the adjacent tissue. A pro-inflammatory milieu is maintained by M1 macrophages and others to declutter the site of injury. This is necessary in order to make room for the consequential anabolic anti-inflammatory progression. The anti-inflammatory milieu is dominated by M2 macrophages that create an environment, which leads to the termination of the inflammation and eventually enables tissue regeneration.

Within the first hours in the course of the inflammatory reaction, surrounding vessels are dilated and vascular permeability is increased by early mediators (Figure 1). In combination with signals released from the early haematoma and the degenerating myofibres, concurrent to the early inflammatory processes, this results in the attraction of circulating inflammatory cells, such as neutrophils [28] and macrophages [29], and different functional subsets of T lymphocytes [30], amongst others. Those primarily recruited macrophages are pro-inflammatory M1 polarized cells that, together with neutrophilic granulocytes, are responsible for phagocytosis. They secrete several cytokines, growth factors, and pro-inflammatory mediators, such as tumor necrosis factor-α (TNF-α), nitric oxide (NO), interleukin-12 (IL-12) and interleukin-1 (IL-1) [27], and interleukin-6 (IL-6). These have a profound impact on the behaviour of adjacent cells and stem cells in regeneration [31]. They also stimulate myoblast proliferation in an IL-6 dependent manner [32]. 

While the M1 phenotype is responsible for phagocytosis and the stimulation of myoblast proliferation, anti-inflammatory M2 macrophages release factors to stop myoblast proliferation and stimulate the subsequent differentiation, fusion and growth of myofibres [33]. M2 polarized macrophages arise from exposure to cytokines such as interleukin-13 (IL-13), interleukin-10 (IL-10), and interleukin-4 (IL-4). After transition from the pro- into the anti-inflammatory healing phase, these alternatively activated M2 macrophages dominate the scene, secreting regulatory cytokines such as TGFβ and IL-10. Understanding the healing cascade becomes more complex due to the additional sub-classification of M2 macrophage; their physiological role varies and their activation differs. M2a are present at later stages of tissue regeneration, M2b often act pro-inflammatory through paracrine effects, and M2c are primarily anti-inflammatory and promote non-myeloid cells. Their specific function in muscle regeneration still remains unclear, but they are known to be responsible for myoblast differentiation [34] as well as for enhancing angiogenesis [35], stimulation of a transient deposition of extracellular matrix (ECM) [32,36], and satellite cell activation [33]. Therefore, they are essential in the early muscle regeneration process [37]. 

On the molecular level, the biosynthesis of pro-inflammatory lipid mediators is gradually altered to anti-inflammatory and pro-resolving lipid mediators. The enzyme COX-2 is responsible for the generation of both pro- and anti-inflammatory molecules at different stages of the process. This becomes relevant when considering that non-steroidal anti-inflammatory drugs (NSAIDs) that are administered as pain medicine target COX-2 and thereby alter the inflammatory reaction of the healing process [38]. 

Regarding the fragile system of development and resolution of inflammation in the early phase of healing the molecular cytokine expression pattern of neutrophils enhances inflammatory cell recruitment, release of proteolytic enzymes as well as reactive oxygen species that can become detrimental for the healing process and may lead to neutrophil induced tissue damage in severe cases [39]. In addition, the macrophage phenotype change in order to resolve the inflammatory process may be hindered and an excessive pro-inflammatory phase showing an enhanced M1 macrophage reactivity might be responsible for a delayed healing and fibrotic development, which clinically leads to scar tissue formation and impaired functional outcomes [34,40,41]. 

### 2.2. Satellite Cells—Multipotent, Muscle Specific Stem Cells

Since the many nuclei of the multinucleated muscle cells called myofibres are terminally postmitotic, under normal conditions, the regeneration of muscle tissue after injury is ensured by multipotent precursor cells called satellite cells. Satellite cells represent the stem cells of muscle tissue and are able to differentiate into myofibers, which build up muscle substance. They are located between the sarcolemma and the basal lamina that surrounds the myofibers. From here, they can be recruited and activated in the event of muscle damage.

Different stages of satellite cell activation, mobilization, and differentiation are necessary to achieve tissue integrity. In the course of regeneration, satellite cells are stimulated to proliferate and differentiate in order to provide myoblasts. 

For approximately three to seven days, the myoblasts proliferate before they differentiate and either fuse with existing (damaged) fibres or fuse to form immature myofibres (myotubes) themselves, creating functional units [31,42]. A minority of satellite cells will return to a quiescent state to provide a sufficient pool of available satellite cells in the newly formed muscle fibers for potential future regenerative needs [43]. 

Satellite cells are sensitive to complement activation, which triggers the subsequent regenerative phases [44]. The key role of satellite cells in healing is broadly discussed in various publications [45,46], as it is known that their depletion completely impedes the regenerative potential in skeletal muscle [47]. The obvious co-occurrence of macrophages and satellite cells is likely, as they cross-stimulate their chemotactic activity by the secretion of soluble factors. Depending on their differentiation, satellite cells can attract monocytes by the expression of MDC (macrophage-derived chemokine), MCP-1 (monocyte chemoattractant protein 1), FKN (fractalkine), and VEGF (vascular endotherial growth factor) [48]. In turn, macrophages produce factors that promote satellite cell proliferation, such as IGFs (insulin like growth factor), TGFβ (transforming growth factor beta), FGFs (fibroblast growth factor), PDGFBB (platelet derived growth factor), EGF (epidermal growth factor), and IL-6 [49,50]. Recent studies show that macrophages directly interact with myoblasts in a direct cell-to-cell contact from the phase of satellite cell recruitment toward growing myotubes [51].

Another putative mediator of muscle regeneration is the connective tissue fibroblast, as it is believed to regulate the satellite cells’ niche. Fibroblasts and satellite cells proliferate near one another after injury, and the absence of fibroblasts leads to premature satellite cell differentiation and the formation of smaller regenerated myofibers [52]. Even though fibroblasts are responsible for the formation of connective tissue fibrosis, the positive reciprocal stimulation of the two cell types underlines another, in a favourable aspect of cellular interdependencies. The ambivalence of fibroblast to either support muscle regeneration or fibrosis is decisive for the healing outcome. PDGF and stem cell factor (SCF) together with mechanical tension induce the formation of proto-myofibroblasts from fibroblasts [53]. These proto-myofibroblasts can then differentiate into myofibroblasts if the concentration of TGFβ, TNFα and thus the inflammatory signalling as well as specialized extra cellular matrix proteins are present [54,55]. Fibroblasts are abundant in healing tissue during the early stages of haematoma maturation [17] and thus during the inflammatory phase of healing. The cytokine pattern during the inflammatory reaction decides the fate of the cells and thus the direction of the healing process toward regeneration or fibrosis. Therefore, the initial inflammatory phase is important in determining the healing outcome [56,57]. 

### 2.3. Interstitial Cells Partake in the Healing Process

During healing, the cross talk between resident muscle and non-muscle interstitial cells with immune cells could play a role in determining the healing outcome. An example is fibroadipogenic progenitors (FAP): these cells are considered as origin of intermuscular adipose tissue. They support myogenic progenitors during muscle fiber maturation, but they also represent a cellular source for fibrosis [58]. Satellite cells as muscle stem cells, FAPs, and immune cells interact in determining the outcome of muscle repair towards regeneration or fibrosis [59]. FAP support toward healthy muscle regeneration seems to depend on the IL4 signaling, a cytokine also involved in supporting the M2/Th2 response that was shown to enhance bone healing [60]. Telocytes, also named interstitial Cajal-like cells or ICLCs, are another cell type found in interstitial muscle tissue. These cells secrete VEGF and thus could play a role during revascularization during healing—a process highly dependent on inflammatory signaling [61]. Cells expressing Abcg2 (ATP-binding cassette sub-family G member 2) are classified as interstitial SP (side population) cells in the muscle. These cells also partake in the repair and regeneration process [62], however, their interdependency with immune cells is still unclear.

## 3. Commonalities and Differences in Healing Cascades of Different Tissues

**Figure 2:**
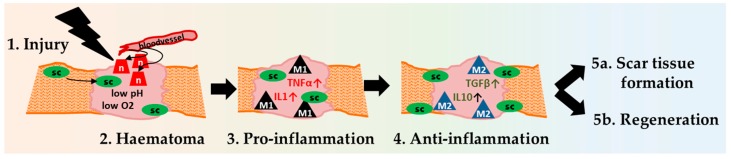
An overview of skeletal muscle regeneration. After an initial trauma, neutrophils (n) and satellite cells (sc) are recruited to the muscle haematoma. Within the haematoma, the pH decreases and the oxygen supply ceases. In the following phase, macrophages (M1) follow and levels of pro-inflammatory cytokine such as TNFα and IL1 increase. In the subsequent anti-inflammatory phase, TGFβ and IL10 are increased, and different sub-populations of macrophages (M2) are active. Finally, healing ends in successful regeneration or in a dysfunctional scar tissue.

Three stages of regeneration can be distinguished in all musculoskeletal tissues: haematoma formation, inflammation, and remodeling (Figure 2). These stages at least partially define the eventual healing outcome [63]. 

**Figure 3:**
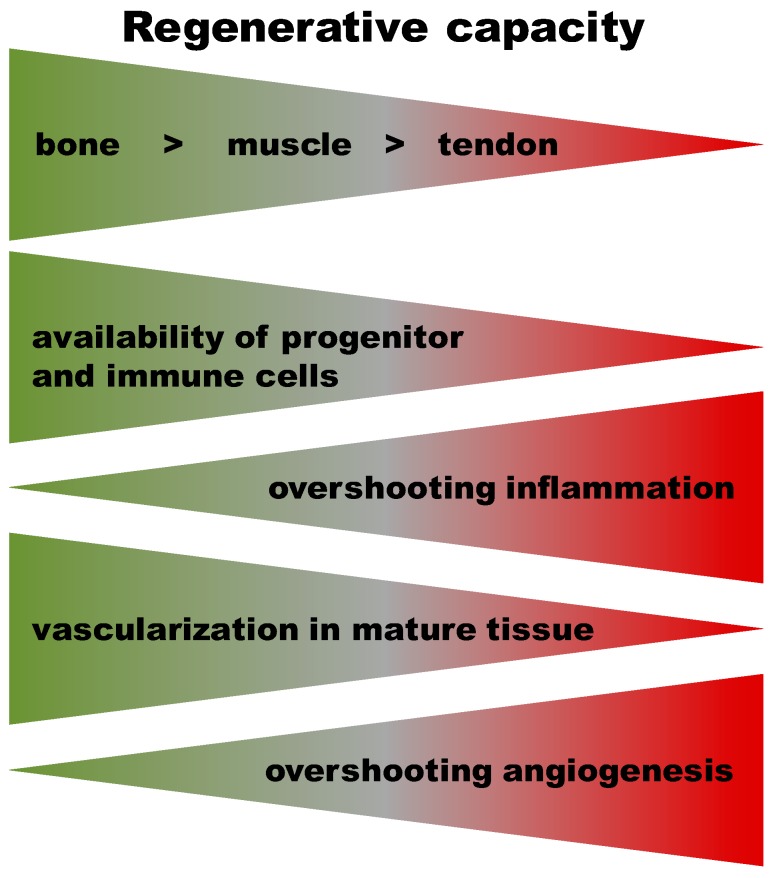
The regenerative capacity differs among musculoskeletal tissues with a high regenerative capacity in bone and a poor one in tendon tissue. The resultant “gradient” of capacity across tissues appears linked to the availability of progenitor cells within the tissues. Stem and progenitor cells reside in the bone marrow and adjacent periosteum/endosteum. They are easily recruited to a bone injury and ready to proliferate and differentiate. They represent a source of important signaling molecules for successful healing (e.g., BMP2). Muscle regeneration after injury relies on interspersed satellite cells and newly detected local stem cells. The available quantities of tissue-residing pluripotent cells appear to be lowest in tendon. The appearance of pluripotent cells post-injury depends on the capacity to form new vessels. After bone injury, revascularization is intense, starting from the periosteum/endosteum and surrounding muscle tissues. To a similar degree, vessel density reappears after muscle injury. The lowest vessel density occurs in tendon, and thus, re-vascularization following injury is a challenge. Surprisingly little is known about the time course of inflammation across these tissues following injury. A pro-inflammatory phase appears to be present across all mesenchymal tissues. In bone, pro-inflammation is followed by distinct anti-inflammation, which seems to be missing or delayed in muscle tissues.

Some elements of the healing cascades in bone, tendon, and muscle tissue are very similar. However, bone has a high regenerative capacity, leading to scar-free healing, whereas tendon and muscle injuries often result in scars. Differences in the regulatory mechanisms during the inflammatory response that initiates healing in muscle, tendon, and bone could shed light on how to prevent fibrosis formation. Particularly, the initial inflammatory phase in regenerating bone could provide a blueprint for an optimal onset to ensure positive healing. The pro-inflammatory signaling after injury is mediated by e.g., TNFα [64], INFγ [65], Ilβ1 and 2 [66] IL6, CXCL1 [67], CXCL4 [68], CD40L [69], SDF1 [70], while anti-inflammatory mediators are for example IL27, HGF [67], TGFβ [71], IL10 and IL22 [72], and IL4 [31]. However, the inflammatory reaction is highly dependent on the tissue environment and the cellular composition and on the time elapsed since injury. Also, the interdependent healing processes have to be considered in the context of the healing sequence. In order to understand the healing capacities of musculoskeletal tissues Figure 3 outlines their differences in the interdependent processes of the healing cascade.

### Early Haematoma, Molecular Interplay, and Differential Regulation in Regeneration

After skeletal muscle trauma, size and extension of the injury appear to be the determining factors as to whether scar or muscle tissue forms. In contrast, tendon trauma often results in the formation of scar tissue with insufficient form and function independent of the extent of injury [73,74,75,76,77]. Another major difference in tissue regeneration is the simple length of the early inflammatory reaction. Three days after injury, the inflammation is already concluded in tendons and the proliferative phase begins. However, muscle inflammation may last for several days, depending on the injury severity, only switching between pro- and anti-inflammation within the first three days [78,79]. In muscle haematoma, there is no stringent regulation of the pro-inflammatory signaling by the occurrence of a distinct anti-inflammatory cascade. In contrast, bone regeneration involves the early up-regulation of anti-inflammatory signals and pro-angiogenic signaling, which keeps pro-inflammation in check [80]. Severe muscle trauma with a subsequent heightened pro-inflammatory reaction can even influence the adjacent bone, altering the inflammatory reaction so that regeneration is impaired [81,82].

A prolonged or disproportionate inflammatory response may lead to an overbalanced angiogenic signalling [83]. Excessive angiogenesis is predominantly present in tendon but to a lesser extend also in muscle and bone. An angiogenic response exceeding that needed for optimal repair leads to unsuccessful wound healing with scar tissue formation [84]. Moreover, completely blocking inflammatory processes in tendon healing and in its reconnection with bone led to impaired healing [85]. Analyses of human shoulder biopsies of rotator cuff tears revealed that the decisive factors for successful healing of the tendon-muscle interplay were balanced inflammation and vascularization; patients with the highest probability of healing showed increased cellular activity, vascularization, and inflammation [86]. 

**Figure 4:**
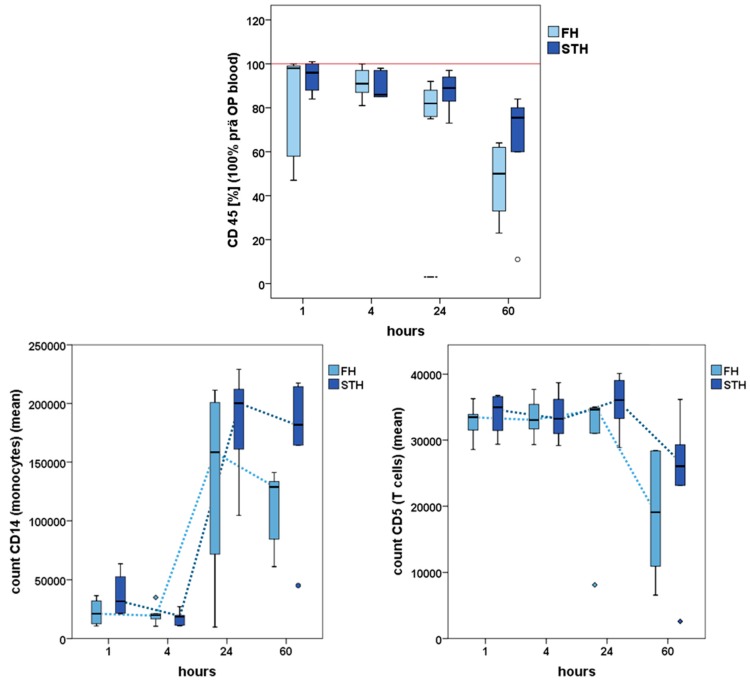
Immune cell percentages in a bone (FH) and a muscle haematoma (STH) over a time course of 60 h: CD45 (leucocytes) percentages are normalized to pre-injury peripheral blood (100%). While being similar to peripheral blood 1 h after injury, leucocytes start to decrease and are clearly lower in bone compared to muscle haematoma at 60 h. Cell counts (normalized to 50,000 leucocytes) show a monocyte increase between 4 and 24 h after injury in bone but a much steeper increase in muscle haematoma. T cell ratios decrease during haematoma maturation, with less T cells in bone compared to muscle haematoma at 60 h, indicating a shift from pro- to anti-inflammation. (Data were acquired with FACS analyses from haematoma generated in sheep: bone haematoma = tibia diaphyseal defect; muscle haematoma = defect in the M. gracilis; *n* = 6 each). Statistic was performed using Mann Whitney U for unpaired samples and Wilcoxon for paired samples, for a descriptive comparison: CD45: FH—1 to 4 h *p* = 0.071, 24 to 60 h *p* = 0.065, STH—24 to 60 h *p* = 0.026; CD14: FH—4 to 24 h *p* = 0.082, STH—1 to 4 h *p* = 0.063, 4 to 24 h *p* = 0.004 60 h; CD5:FH—24 to 60 h 0.041, STH—24 to 60 h *p* = 0.026.

A sheep study comparing early haematoma in bone and muscle revealed that during the first 60 h of healing, the immune cell composition in both tissues is different (Figure 4, unpublished data). Even though the early pro-inflammatory reaction appears similar in bone and muscle tissues with cell quantities being comparable 1 h after injury, inflammation in regenerating bone is already retreating while it is still ongoing in muscle.

**Figure 5:**
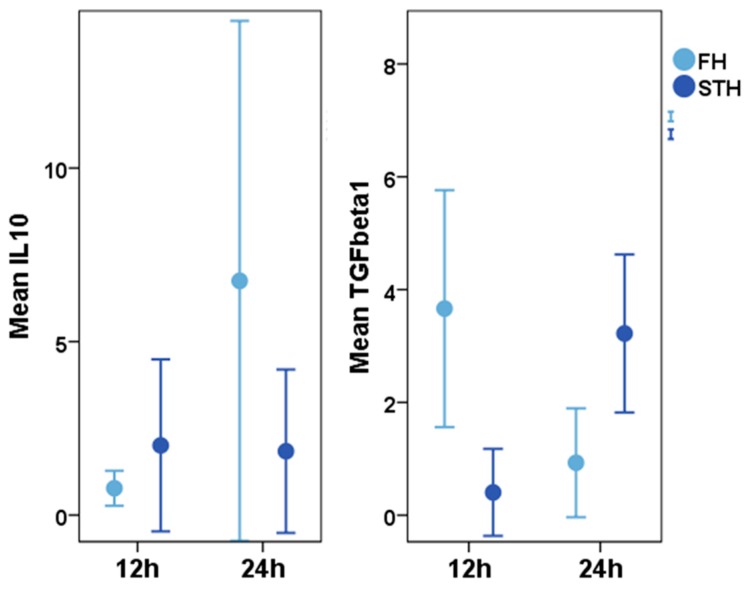
Expression values for IL-10 (interleukin 10) and TGF-β1 (transforming growth factor beta 1) (median values) in a haematoma in the diaphyseal part of sheep tibia (bone haematoma = light blue) and in the gracilis muscle (without any contact to bone/muscle haematoma = dark blue) during the first 24 h after injury. Cytokine expression was measured 12 and 24 h after injury. *n* = 6. Using Mann Whitney U for paired and Wilcoxon for unpaired samples, a descriptive statistical analyses revealed an increase of IL10 in the FH *p* = 0.041 and a decrease of TGFβ1 *p* = 0.015; within the STH, no increase of Il10 was monitored while TGFβ1 increased *p* = 0.009, different expression pattern within the FH and STH lead to a difference in TGFβ1 expression at 24 h after injury *p* = 0.031. For more details, please refer to [16].

Expression analyses of the anti-inflammatory factor IL-10 revealed up-regulation of this cytokine after 24 h in bone haematoma, which was not seen in muscle haematoma (Figure 5) [16]. Early up-regulation of IL-10 indicates that the pro-inflammatory reaction, which is essential during the early healing processes, is halted to prevent it from turning into a detrimental process during later regeneration phases. Yet, the apparently controversial regulation could also be due to previously described paradoxical effects, in which an immunosuppressive as well as an activating function has been attested to IL-10 [87].

TGFβ is a key player and pluripotent cytokine in the local immune environment. The TGF β members were discovered in the 1980 and named transforming growth factors due to their cell transforming properties [88]. They are still under consideration today as deciding factors in the fate of muscle healing towards regeneration or fibrosis. TGFβ1 is known as the most potent pro-fibrogenic factor and regulator of fibrosis [89]. On the other hand, TGFβ1 also affects extracellular matrix degradation as a regulator of expression of metalloproteinase (MMPs) and their inhibitors (TIMPs) [90]. Muscle regeneration is decisively improved by anti-fibrotic agents that block or inactivate TGFβ1, such as interferon, relaxin, or suramin [91,92,93]. Reduction of fibrosis can also be achieved by inhibiting TGFβ1 by platelet-rich plasma [94] or by application of insulin-like growth factor-1 (IGF-1) [95]. The inhibition of TGFβ1 thus impacts myoblasts, fibroblasts, and inflammatory cells during muscle regeneration [96,97]. 

In general, TGFβ actively participates in the regulation of inflammation by having an essential function in the maintenance of homeostasis and T-cell signaling [98] and is secreted, together with IL-10, from CD4+ as well as from CD8+ regulatory T cells [99]. Moreover, the ratio between the cytokines TGFβ1, TGFβ2 and TGFβ3 is important for the healing outcome [100]. Changing the ratio of these cytokines in favour of TGFβ3 results in less scar tissue formation in skin wound healing [101]. 

Autocrine and paracrine muscle-growth inhibition has previously been attributed to myostatin, which is a TGFβ family member that is most dominantly expressed in skeletal muscle [102]. TGFβ1 and myostatin are released by muscle cells to provide signaling cues for muscle regeneration [103]. In an animal model of chronic muscle disease, myostatin inhibition increased muscle growth and reduced fibrosis, indicating direct promotion of fibroblast proliferation resulting in muscle fibrosis in vivo [104]. Muscle fiber repair improved after anti-myostatin treatment in a diabetic mouse model of limb ischemia [105]. Recently, the importance of myostatin for bone regeneration has been considered [106]. Myostatin inhibition restored bone healing in diabetic animals [107]. Current resear proved that inflammation is a trigger for the interaction of myostatin in musculoskeletal injury. A myostatin inhibitor that was activated upon inflammation proved beneficial in regenerating functional skeletal muscle tissue [108].

TGFβ1 is differentially regulated during the initial phase of healing in bone and muscle (Figure 5), with an early increased expression leading to regeneration in bone and a later upregulation resulting in scar tissue formation in muscle. TGFβ1 expression already decreases in the bone haematoma 24 h after injury, whilst its expression in the muscle haematoma is still increasing in that time frame [16].

The higher regenerative capacity of bone may hence also be due to differentially regulated cytokine expression. Thus, stringent regulation of the early inflammatory reaction and angiogenic processes might optimize muscle healing toward “*restitutio ad integrum*,” as seen in bone. 

**Figure 6:**
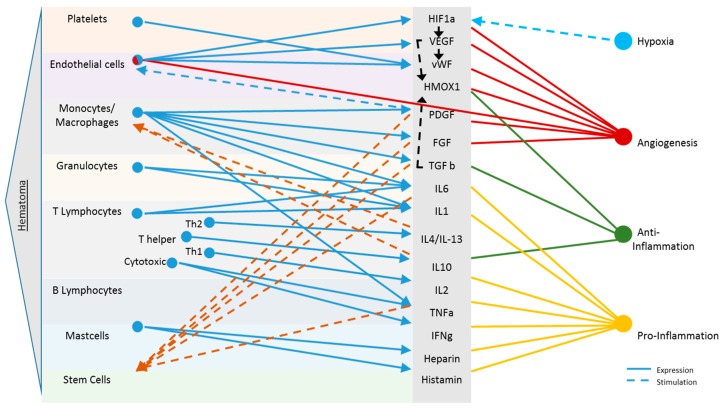
Molecular and cellular signaling in haematoma of the musculoskeletal system. Following each injury, upon disruption of blood vessels, haematoma formation presents the starting point of any healing process. Cells present in the musculoskeletal haematoma express a complex mixture of cytokines (solid blue lines). These are key regulators in initial regenerative processes such as hypoxia (light blue), angiogenesis (red) and inflammation (anti-inflammation: green, pro-Inflammation: yellow). The present milieu and cytokine cocktail again stimulate specific cells (e.g., stem cells, monocytes/macrophages and endothelial cells), respectively, to further promote regeneration (dashed lines). Only selected cells and cytokines present during this early healing phase are depicted [16,18,65,109,110,111,112,113,114,115].

Another influencing factor for the regenerative capacity of tissues is the types of cells that are naturally present near the injury, which provide a valuable source of progenitors and cytokines during healing. Interacting immune cell subsets may remain the same, but their ratio and activity are highly influenced by surrounding tissue. Depending on the tissue, the local macrophages differ in number (more in spleen than muscle), amount of vessels (very low in tendons), and most importantly, the types and local concentrations of stem cells (bone marrow provides the best source). Figure 6 overviews these cellular interactions. 

## 4. Myoimmunology-Based (Cell) Therapeutic Options and Future Directions

Most patients with musculoskeletal injuries are treated with non-steroidal anti-inflammatory drugs (NSAIDs) for pain. However, these drugs can impair healing by down-regulation of the inflammatory COX2 pathway, as summarized for muscle and bone injuries [116] and suggested in the context of bone-tendon healing [117]. Thus, new therapies, particularly those addressing immune modulatory strategies, require a profound understanding of the role of early inflammation in regeneration. It also illustrates that much can be gained from a basic understanding of the early inflammatory cascade and how this understanding can directly impact daily treatment such as re-visiting our current patient medication strategy. 

In order to regenerate severe skeletal muscle trauma, several therapeutic options have to be discussed. An option is the usage of cell-based therapies, either focusing on the cells’ own actions, their paracrine effects, or their interaction with other cell types. Aiming at the early healing phase for corrective interference, current approaches that proved to be successful in improving compromised bone healing should be considered as possible approaches for muscle tissue regeneration using bone tissue as a blueprint for regeneration in order to reduce the higher scar tissue formation potential due to prolonged muscular inflammatory signalling. Some initial success in improving bone healing through cell based immune modulation has been reported [117,118]. However, for muscle regeneration, these approaches are still, though highly promising, in the fledgling stage. 

B and T lymphocytes are well known to steer and influence bone healing processes [65,109,119,120]. Considering that specific CD8 T-cell subset levels in peripheral blood can even serve as a biomarker to elucidate a patients probability of healing after bone injury [65], this specific pro-inflammatory T-cell population might also be of interest in muscle regeneration and diagnostics. Meanwhile, anti-inflammatory regulatory T-cells (Tregs) have been shown to regulate the general frequency of other T cell populations, as well as the myeloid cell population that infiltrates the injured tissue in an immediate response. Tregs contain the number of myeloid cells and promote their switch from a pro- to an anti-inflammatory phenotype [31,121]. Moreover, in a mouse model, Tregs were shown to be predominantly present in close proximity to regenerating fibre after intramuscular cardiotoxin administration, suggesting a direct impact on those cells that are responsible for local tissue repair [121]. Recent studies revealed that an age-dependent lack of Tregs causes poor repair of skeletal muscle in old mice [122]. Modulation of pro-inflammatory and anti-inflammatory lymphocyte ratios such as T-cell (CD8+ T-cell/Treg) or macrophage (M1/M2) ratios or their respective cytokine expression patterns in order to control the inflammatory environment by favouring its resolution would be a potential strategic approach to foster regeneration.

There are obvious analogies in bone and muscle tissue healing regarding the very early cellular haematoma composition [16]. Thus, an application of CD31+ cells appears feasible to circumvent scar tissue formation in muscle. These cells’ have demonstrated potential in bone regeneration via synergistic angiogenic and immune modulatory effects. In a rat model for impaired fracture healing, the local application of CD31+ cells enriched from peripheral blood led to significantly reduced amounts of fibrotic tissue in the fracture gap [118], presumably due to an early milieu modulation towards anti-inflammation. 

The induction of M2 macrophages coincides with the termination of pro-inflammatory processes, and various studies describe the switch from pro- to anti-inflammatory status during muscle regeneration [33,123]. Research is therefore currently focussing on the possibility of using an artificially induced cell population or phenotypic switch in M1 to M2 macrophages to enhance regeneration in muscle [122,124]. A timely application of alternatively activated macrophages (M2) or the creation of a cytokine or growth factor driven milieu supporting either the recruitment of anti-inflammatory cells or anabolic, anti-inflammatory processes should be taken into consideration. In the context of liver fibrosis, Thomas et al. [125] reported that the infusion of bone marrow derived macrophages into the hepatic portal vein of mice after liver injury resulted in the upregulation of MMPs 9 and 13, which participate in the process of hepatic scar tissue remodelling, and led to paracrine effects via elevated anti-inflammatory IL-10 levels. IL-10, again, is well known to promote M2 differentiation of macrophages.

Mesenchymal stromal cells (MSCs) have also drawn interest in research on the regeneration of various human tissues, especially skeletal muscle [126,127,128]. MSCs show a unique expression of surface antigens and could potentially differentiate toward adipogenic, chondrogenic, and osteogenic cells and also toward myogenic cells [129,130]. In addition, there have been recurring debates on whether MSCs are actually derived from pericytes [131,132,133]. Pericytes partake in modifying the local immune response upon muscle injury, and lineage tracing has indicated their direct contribution to myofiber regeneration [134].

To test the impact of MSCs on functional muscle force, a rat model of crush trauma to the musculus soleus was established, and muscle force was analyzed during regeneration over time [135,136]. Skeletal muscle regeneration and muscle strength improved significantly after injection of autologous bone marrow MSCs (bm-MSCs) in rats [137]. The transplanted bm-MSCs could be visualized within the target area and fusion events with regenerating myofibers were observed [138]. The positive effects of transplanted bm-MSCs were dose-dependent, with the largest amount of transplanted cells leading to the most pronounced improvement in muscle force [139] in both sexes [136]. The great therapeutic potential of bm-MSCs in muscle regeneration was confirmed, as muscle contraction force recovered more quickly after their transplantation into the muscle tissue (either directly post trauma or after a delay) [140]. This suggests that bm-MSCs positively influence the healing cascade at different time-points of the regenerative process. 

The preclinical data have been successfully translated into a clinical trial conducted at Charité University Medical School Berlin, where patients receiving placenta-derived stem cells after total hip replacement surgery produced significantly higher isometric contraction force than those receiving a placebo [141]. However, the positive effect plateaued at medium cell concentrations. MSCs embedded in a porous alginate scaffold positively influenced muscle regeneration after crush muscle trauma [142]. Since the carrier material was transplanted next to the injury, the effects can be contributed to paracrine effects from the cells. However, the question remains of whether the effect was achieved by a direct paracrine stimulus to the satellite cells to build myofibers or by encouraging the myofibroblasts to lay down an adequate matrix for satellite cell differentiation. It is also possible that the MSCs influenced the inflammatory environment or macrophages, which highly stimulated regeneration via the mentioned cascade. The best healing outcome was achieved when the cells were transplanted in combination with VEGF and IGF, but the growth factors alone had little effect. In bone, the highest physiological VEGF levels occur at the switch between pro- and anti-inflammatory phases [16]. Thus, the provided growth factors could possibly signal MSCs to take immediate anabolic action. 

These cell therapeutic options aim to reduce the pro-inflammatory effect during muscle healing which would decrease the regenerative healing potential of the muscle and further the formation of fibrotic tissue. These therapies would not only be supportive of muscle regeneration but also on bone regeneration which was used as a blue print for these myoimmunological approaches as they have already been proven to gain positive results in bone healing. 

## 5. Concluding Remarks

While the role of inflammation in regeneration is gaining more and more attention, little is known on how the initial inflammation following skeletal muscle trauma interacts with local regenerative cascades in a host tissue and on how trauma extent and severity determine the fate of the healing response. Recent research efforts have revealed a uniquely orchestrated cellular reaction accompanied by paracrine signalling of muscle and immune cells. In this review we have pointed out that considering similarities and differences in healing cascades of the musculoskeletal tissue types can help to understand varying healing outcomes and that it could be beneficial to copy principles from other tissues in order to artificially but endogenously simulate and initiate positive processes. Such control of the inflammatory milieu can lead to the regeneration of functional muscle, e.g., using bone as a regeneration-blueprint while learning from tendon’s scar formation tendencies. This provides a fascinating and yet essential basis for the development of future therapeutic approaches that aim at enhancing healing toward reconstitution of form and function.
